# Building genomic resources to facilitate the study and use of *Solanum microdontum,* a wild relative of cultivated potato

**DOI:** 10.1093/g3journal/jkaf253

**Published:** 2025-10-23

**Authors:** Anne Frances Jarrell, John P Hamilton, Joshua C Wood, Brieanne Vaillancourt, Jessica Norling, David Douches, Carol Robin Buell

**Affiliations:** Institute for Plant Breeding, Genetics and Genomics, University of Georgia, Athens GA 30602, United States; Center for Applied Genetic Technologies, University of Georgia, Athens GA 30602, United States; Department of Crop and Soil Sciences, University of Georgia, Athens, GA 30602, United States; Center for Applied Genetic Technologies, University of Georgia, Athens GA 30602, United States; Center for Applied Genetic Technologies, University of Georgia, Athens GA 30602, United States; Department of Plant, Microbial and Soil Sciences, Michigan State University, East Lansing, MI 48824, United States; Department of Plant, Microbial and Soil Sciences, Michigan State University, East Lansing, MI 48824, United States; Institute for Plant Breeding, Genetics and Genomics, University of Georgia, Athens GA 30602, United States; Center for Applied Genetic Technologies, University of Georgia, Athens GA 30602, United States; Department of Crop and Soil Sciences, University of Georgia, Athens, GA 30602, United States; The Plant Center, University of Georgia, Athens, GA 30602, United States

**Keywords:** *Solanum microdontum*, potato, diversity, disease resistance

## Abstract

*Solanum microdontum* Bitter is a diploid wild Andean relative of potato that has shaped the domestication and adaptation of modern cultivated potato to diverse environments. *S. microdontum* has the potential to provide a wealth of untapped genetic material for use in addressing current challenges in potato breeding. Here, we report a high-quality 772 Mb reference genome sequence for *S. microdontum* that is anchored to 12 chromosomes. The resulting genome assembly has 99.0% complete benchmarking universal single copy orthologs and an N50 scaffold length of over 57 Mb, indicating a high level of completeness. Annotation of the assembly resulted in the identification of 37,324 protein-coding genes and 65% repetitive sequence. A total of 1,187 nucleotide-binding leucine-rich repeat genes were predicted from the assembly, of which, 93.1% overlapped an annotated high-confidence gene model. A *k*-mer-based kinship matrix derived from a 107-member *S. microdontum* diversity panel revealed an underlying population structure that corresponds to geographic proximity. The *S. microdontum* dataset enhances publicly available potato genome resources by providing breeders with genetic, molecular, and germplasm resources for newly developed diploid potato breeding programs.

## Introduction

*Solanum tuberosum* L., commonly known as the potato, is a tuberous crop that is a staple food crop across the globe. Currently, it is the world's third most important food crop in terms of consumption, behind only wheat and rice (Devaux et al. 2014). Potato also delivers more edible biomass and caloric energy per acre than any other food crop (Narváez-Cuenca et al. 2018). Potato was domesticated 8,000–10,000 years ago in the Andes in South America and brought to Europe during the Columbian exchange. Today, potatoes are cultivated on every continent except Antarctica, due in part to their adaptability. The remarkable diversity of potatoes is reflected in their germplasm resources, which include over 100 recognized wild and cultivated species (Spooner et al. 2014). While many landraces and wild relatives are diploids, cultivated potato is an autotetraploid; however, the majority of these diploid relatives can hybridize with cultivated potato either directly or through techniques that overcome ploidy barriers (Hanneman 1989; Camadro 2010). Historically, potato landraces and wild relatives have played an important role in contributing adaptive traits to cultivated potato varieties (Rieman et al. 1954; Plaisted and Hoopes 1989; Jansky et al. 2013).

*Solanum microdontum* Bitter is a diploid wild species of potato native to the Andes mountains, specifically from Northern Bolivia to Northwestern Argentina (van den Berg and Spooner 1992). *S. microdontum* is found across a wide range of altitudes, ranging from 1300 to 3500 m above sea level, and throughout a broad array of habitats, including humid forests, rocky slopes, sandy soil, and marginal lands (Correll 1962). Though the tubers are small, *S. microdontum* has several traits of interest to potato breeders, most importantly, disease resistance. *S. microdontum* is resistant to pathogens such as late blight (*Phytophthora infestans* (Mont.) de Bary), soft rot (*Dickeya dianthicola* Samson), and Fusarium dry rot (*Fusarium sambucinum* Fuckel) (Lynch et al. 2003; Bisognin et al. 2005; Ma et al. 2022; Fenstemaker et al. 2023). Introgressions from *S. microdontum* are present in the genome of cultivated potato (Hardigan et al. 2017), which are hypothesized to have been introduced to cultivated potato as it moved south from the center of domestication through the native range of *S. microdontum*. One introgression of particular importance is an allele at the *StCDF1* locus, which enables tuberization under long-day conditions. Its introgression allowed for the expansion of potato cultivation outside its near-equatorial domestication center, including Europe and other latitudes with long summer days (Hardigan et al. 2017).

Further introgression of adaptive traits from wild potato relatives such as *S. microdontum* has become more feasible with the recent emergence of efforts to breed potatoes at the diploid level (Jansky et al. 2016). This process involves crossing tetraploid lines to a haploid inducer line, which creates dihaploid (diploid) progeny. Fertile and self-compatible diploid lines can be selfed to generate inbred lines and then crossed with other inbreds to create an F1-hybrid, a breeding scheme analogous to hybrid maize or rice breeding. This would hasten breeding efforts in potato by simplifying segregation ratios, allowing for easier purging of deleterious alleles, and enabling the development of inbred lines to incorporate genes and traits into a fixed genetic background. Thus, introgression of adaptive traits from wild potato relatives such as *S. microdontum* would be greatly facilitated (Jansky et al. 2016; Su et al. 2020).

Here, we report a de novo reference genome for the wild potato relative *S. microdontum* as well as a sequenced diversity panel of 107 *S. microdontum* accessions to facilitate its use as a donor of adaptive alleles, particularly in the context of diploid potato breeding programs. Using PacBio HiFi long reads in conjunction with Hi-C reads, we constructed a high-quality chromosome-scale reference genome for *S. microdontum* that encoded 37,324 genes. Whole genome sequencing of a 107-member *S. microdontum* diversity panel revealed population structure that correlates with geographic origin. These resources will be useful for comparative genomics studies between *S. tuberosum* and *S. microdontum*, pinpointing genes underlying adaptive traits in *S. microdontum*, and identifying accessions to be used for introgression.

## Methods and materials

### Plant materials

The *S. microdontum* diversity panel is comprised of 107 accessions from the USDA-ARS GRIN collection (Supplementary Table 1). Seeds were germinated, and a single plant was selected from each to represent that accession. Plantlets for each were maintained in tissue culture on Murashige and Skoog medium (Murashige and Skoog 1962). For tissue collection, plants were grown in a growth chamber under a 12-h photoperiod at 500 µmol/m²/s light intensity with 75% relative humidity and 25 °C day/18 °C night temperature, ramping up to maximum temperature and ramping down to minimum temperature over 3.5 h. The genome size of 3 *S. microdontum* accessions (PI 310979, PI458355, and PI 498123) was determined using flow cytometry at the Flow Cytometry and Imaging Core laboratory at the Virginia Mason Research Center (Seattle, WA). The late blight-resistant reference accession PI 595506 was chosen from the diversity panel through a series of late blight detached leaf greenhouse bioassays using 2 *Phytophthora infestans* isolates US-23 and NL13316 and whole plant greenhouse bioassays using isolate US-23, following the procedures outlined by Karki et al. (2021).

### DNA isolation, library preparation, and sequencing

For the PI 595506 reference genome, immature leaves from a single 3.5-week-old plant were collected and ground in a mortar and pestle with liquid nitrogen. High-molecular-weight DNA was isolated using the NucleoBond HMW DNA kit (Düren, Germany). DNA libraries were prepared using the PacBio Template Prep Kit 3.0 (Menlo, CA) and sequenced on a PacBio Revio instrument by the University of Minnesota Genomics Center. Whole immature leaves from a 4-week-old plant of the reference accession were used to generate 2 Hi-C libraries using the Phase Genomics Proximo Kit version 4.5 (Seattle, WA) and sequenced on an Illumina NovaSeq 6000 and an Illumina NovaSeq X Plus in paired-end mode to 150 nt (Supplementary Table 2).

For the diversity panel, tissue culture plantlets or plants grown in the growth chamber were used for total DNA isolation. Tissue culture plantlets with a relatively high proportion of leaf tissue versus stem tissue were chosen for sampling. Growth chamber plants were sampled between 4 and 9 weeks of age using a leaf punch, prioritizing young leaves. In both cases, tissue was flash frozen in liquid nitrogen immediately after harvest and ground with liquid nitrogen using a mortar and pestle. DNA was isolated from the diversity panel using a Promega Wizard® Genomic DNA Purification Kit (Madison, WI). For a subset of accessions, the Promega Wizard kit failed to yield sufficient quality DNA, and thus, a modified Sorbitol-CTAB DNA extraction protocol (Tel-zur et al. 1999) was used. Libraries were prepared using the PerkinElmer NEXTFLEX Rapid XP DNA-Seq Kit HT (Lafayette, CO) and sequenced via whole genome shotgun sequencing on an Illumina NovaSeq 6000 instrument in paired-end mode to 150 nt by the Texas A&M Genomics & Bioinformatics Service (Supplementary Table 2).

### RNA isolation, library preparation, and sequencing

Tissue was collected in triplicate from the leaf, open flower, stem, stolon, root, and tuber of PI 595506 for Oxford Nanopore Technologies (ONT) full-length cDNA sequencing. Tissue was flash frozen in liquid nitrogen and ground using a mortar and pestle. Below-ground tissue was quickly rinsed of soil and dried as needed before flash freezing. Total RNA was isolated using a hot borate protocol (Wan and Wilkins 1994) and any residual DNA removed using the Invitrogen TURBO DNA-free™ Kit (Vilnius, Lithuania). Poly-A selection was performed using an Invitrogen Dynabeads™ mRNA Purification Kit (Vilnius, Lithuania), and replicates were pooled for library preparation. cDNA libraries (Supplementary Table 2) were prepared using the ONT cDNA-PCR sequencing library preparation kit (SQK-PCS111) (Oxford, England), loaded onto FLO-MIN106 flow cells with R9.4.1 pore chemistry, and sequenced on MinION sequencers. Reads were basecalled with Guppy (v6.5.7) (https://nanoporetech.com/software/other/guppy) using the following parameters: –config dna_r9.4.1_450bps_sup.cfg –trim_strategy none –calib_detect.

### Genome assembly and quality assessments

GenomeScope (Ranallo-Benavidez et al. 2020) (v2.0) was used to assess heterozygosity of the reference accession using Illumina reads at a *k*-mer length of 21. PacBio HiFi reads were assessed for quality using NanoComp (De Coster and Rademakers 2023) (v1.1.0), filtered to remove reads less than 15 kb, and subsampled to 48× coverage using SeqKit (Shen et al. 2016). Hi-C reads were assessed for quality using Phase Genomics’ hic_qc script (phasegenomics 2024) (v2.5.1). The reference genome was assembled using hifiasm (Cheng et al. 2021) (v0.24.0) with both HiFi reads and Hi-C reads used as input to enhance assembly contiguity and completeness. In preparation for scaffolding, Hi-C reads were subsequently aligned to the assembly with BWA-MEM (Li 2013) (v0.7.18) using the −5SP option, PCR duplicates were removed using samblaster (Faust and Hall 2014) (v0.1.26), and the resulting SAM file was converted to a BAM file using SAMtools (Danecek et al. 2021) (v0.1.20). The alignment BAM file and assembly were input into YaHS (Zhou et al. 2023) (v1.1) to scaffold the assembly. Juicer Tools (Durand et al. 2016b) (v1.9.9) was used to prepare Hi-C contact maps for visualization. Manual curation of the assembly to reorient and correct the resulting pseudomolecules was performed using Juicebox (Durand et al. 2016a) (v1.11.08). Contigs less than 50 kb were filtered out after scaffolding and curation, and the final assembly was checked for completeness using Benchmarking Universal Single Copy Orthologs (BUSCO) (Manni et al. 2021) (v5.8.3). *k*-mer spectrum comparisons were made using the KAT (Mapleson et al. 2017) (v2.4.2) comp function with a *k*-mer size of 31. A LTR Assembly Index score was calculated from the results using LTR_retriever (Ou et al. 2018) (v3.2).

### Genome annotation

The genome assembly was repeat masked by first creating a custom repeat library using RepeatModeler (Smit et al. 2008) (v2.03). Protein-coding genes were then filtered out from the initial repeat library using ProtExcluder (Campbell et al. 2014) (v1.2). The initial repeat library was then combined with Viridiplantae repeats from RepBase (Bao et al. 2015) (v20150807) to generate the final custom repeat library, and the genome assembly was repeat-masked using RepeatMasker (Smit et al. 2013–2015) (v4.1.2-p1) with the parameters -e ncbi -s -nolow -no_is -gff.

ONT cDNA reads were checked for quality using NanoComp (De Coster and Rademakers 2023) (v1.23.1). The ONT cDNA reads were processed with Pychopper (epi2me-labs 2024) (v2.7.10), and trimmed reads greater than 500 nt were aligned to the genome using minimap2 (Li 2018) (v2.26-r1175) with a maximum intron length of 5,000 nt. RNA-seq data from *S. microdontum* leaf tissue were also downloaded from the NCBI Sequence Read Archive (SRX11438934) (Bhatia et al. 2023) and assessed for quality using FastQC (Andrews 2010) (v0.12.0). The RNA-seq library was processed with Cutadapt (Martin 2011) (v4.6) using a minimum length of 100 nt and quality cutoff of 10 then aligned to the genome using HISAT2 (Kim et al. 2019) (2.2.1). The aligned RNA-seq and ONT cDNA reads were each assembled using Stringtie (Shumate et al. 2022) (v2.2.1), and transcripts less than 500 nt were removed.

Initial gene models were created using BRAKER2 (Stanke et al. 2008) (v2.1.6) using the soft-masked genome assembly and the aligned RNA-seq library. The gene models were then refined using 2 rounds of PASA2 (Haas et al. 2003) (v2.5.2) to create a working gene model set. High-confidence gene models were identified from each working gene model by filtering out gene models without expression evidence or a PFAM domain match and those that were a partial gene model or contained an interior stop codon. Functional annotation was assigned by searching the working gene model proteins against the TAIR (Lamesch et al. 2012) (v10) database and the Swiss-Prot (Schneider et al. 2005) plant proteins (release 2024_05) database using BLASTP (Camacho et al. 2009) (v2.12.0) and the PFAM (Mistry et al. 2021) (v37.0) database using PfamScan (Mistry et al. 2007) (v1.6). Annotation was assigned based on the first significant hit. The annotation was checked for completeness using BUSCO (Manni et al. 2021) (v5.8.3) (Supplementary Table 3). Whole-genome de novo transposable element annotation was performed using EDTA (Ou et al. 2019) (v2.2.2), providing the genome assembly and the high-confidence gene model coding sequences.

### NLR gene prediction and analyses

NLR genes were predicted from the *S. microdontum* genome assembly with NLR-Annotator (Steuernagel et al. 2020) (v2.0) using the motif files provided on the NLR-Annotator GitHub (Steuernagel 2022). Assemblies of *Arabidopsis thaliana* TAIR10 (Lamesch et al. 2012), tomato (*Solanum lycopersicum*) var. Heinz 1706 vSL4.0 (Hosmani et al. 2019), and the cultivated potato (*S. tuberosum*) reference assembly DM v6.1 (Pham et al. 2020) were used as controls with known numbers of previously identified NLR genes to confirm the utility of this tool, especially in Solanaceous crops. Predicted genes were overlapped with the representative working gene models and representative high-confidence gene models using BAMTools intersect (Barnett et al. 2011) (v2.5.2). An NLR local synteny plot was created with pyGenomeViz (Shimoyama 2024) (v1.0.0) by providing start/end coordinates for representative high-confidence gene models that overlapped predicted NLR genes as well as surrounding genes in the region of interest.

### Genome synteny

Seven wild potato species were chosen for synteny analysis based on the presence of a chromosome-scale publicly available genome assembly. The assembly, annotation, and peptide sequence for each were downloaded from various repositories (Supplementary Table 4) and analyzed with the SeqKit (Shen et al. 2016) (v2.9.0) stats function for quality control. These were included alongside the *S. microdontum* genome and the DM v6.1 reference genome for analysis with GENESPACE (Lovell et al. 2022) (v1.2.3). OrthoFinder (Emms and Kelly 2019) (v2.5.4) was used to determine orthogroups, make orthologous gene trees, and infer the species tree using STAG and STRIDE algorithms (Emms and Kelly 2017). The resulting species tree was colored according to clade membership based on earlier taxonomic classifications in the literature (Spooner and Castillo 1997; Spooner et al. 2018; Huang et al. 2019).

### *S. microdontum* population genetic analyses

Publicly available sequencing data from the DM reference genome (Pham et al. 2020) were also included in the diversity panel as a cultivated comparison (NCBI accession SRR11908546). Raw reads were assessed for quality using FastQC (Andrews 2010) (v0.11.9), trimmed using Cutadapt (Martin 2011) (v4.5), and aligned to the draft reference genome using BWA-MEM (Li 2013) (v0.7.17) to check the percentage of multimapping reads. The kmerGWAS (Voichek and Weigel 2020) (v3.2.2) pipeline was used to generate a kinship matrix from the trimmed reads using a *k*-mer length of 31, a minimum *k*-mer appearance rate of 2, and a minor allele frequency of 0.05.

The kinship matrix was visualized directly in the form of a heatmap using R package pheatmap (v1.0.12) and used to create a dendrogram using R package pvclust (v2.2-0) and a PCA plot using base R function prcomp. Population structure was determined by setting a threshold of relatedness (kinship = 0.768) and grouping individuals who all share a kinship value above the threshold, either directly or indirectly, using R package igraph (v2.1.4). Groups with less than 5 individuals were not considered. Collection coordinates for each PI were downloaded from USDA-GRIN and visualized using R package sf (v1.0-19) for plotting spatial data. Points were colored according to membership in the 6 largest population groups.

## Results and discussion

### Genome assembly of *S. microdontum*

**Fig. 1.**
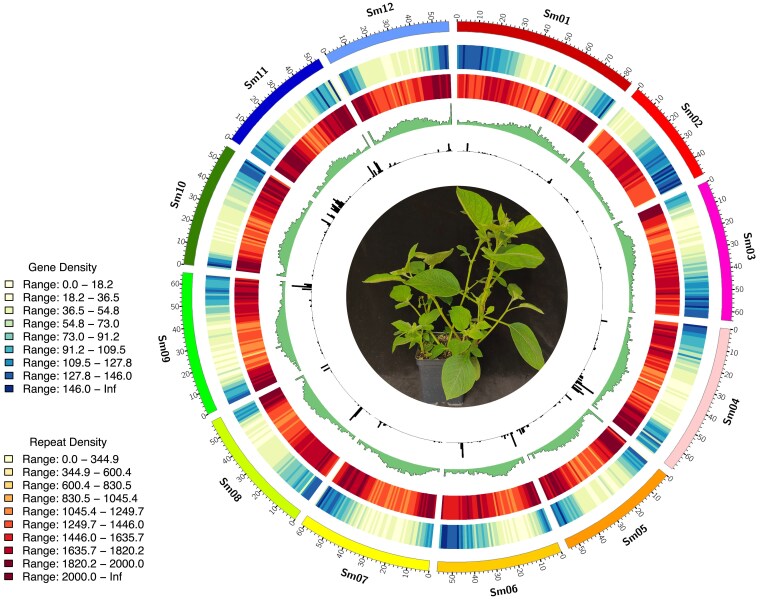
*Solanum microdontum* genome sequence. Tracks from outside to inside: 12 chromosomes in Mb, gene density (see color legend), total repetitive content density (see color legend), GC content (%) (green), NLR gene content (black). All data evaluated in 1Mb bins.

*S. microdontum* accession PI 595506 was chosen as the reference accession based on its late blight resistant phenotype as demonstrated through a series of late blight detached leaf and whole plant greenhouse bioassays (Norling and Douches, in prep.). Flow cytometry of 3 *S. microdontum* accessions revealed an estimated 1C value of 795 Mb (Supplementary Table 5). GenomeScope analysis of Illumina short reads of the reference accession revealed a heterozygosity rate of 1.19%, similar to that of other wild diploid potato species (Hirsch et al. 2013; Aversano et al. 2015; Tang et al. 2022) (Supplementary Fig. 1). Thus, we employed PacBio HiFi sequencing to generate 91 Gb of high-fidelity reads, an estimated 108 × coverage, with an N50 read length of 20,616 bp. Filtered reads were assembled along with 88.7 Gb of Hi-C reads using hifiasm to produce a draft assembly of length 778,965,393 bp with an N50 contig length of 40,218,693 bp (Supplementary Table 6). Hi-C reads were used to scaffold the assembly using YaHS, and after inspection, manual correction, and a 50 kb filtering step, the assembly was finalized (Fig. 1, Supplementary Fig. 2). The resulting assembly contains 12 chromosomes and is composed of 333 scaffolds with an N50 scaffold length of 57,971,928 bp (Table 1, Supplementary Table 6). The total assembly length is 772,092,446 bp, consistent with our previous flow cytometry genome size estimates for *S. microdontum* (Table 1, Supplementary Table 5). An LTR Assembly Index (LAI) score of 13.09 was achieved, indicative of a reference-quality assembly (Ou et al. 2018).

**Table 1.** *Solanum microdontum* genome assembly metrics.

Metric	Number
Number of scaffolds	333
Total assembly size (bp)	772,092,446
Assembly size in chromosomes (bp)	730,521,879
% sequence in chromosomes	94.616
Min. scaffold length (bp)	50,214
Mean scaffold length (bp)	2,318,596
Max. scaffold length (bp)	84,553,384
L50 scaffold count	6
L90 scaffold count	12
N50 scaffold length	57,971,928
# Ns	8,400
%N	0.001
%GC	34.91

To further assess the quality of the assembly, whole-genome paired-end sequencing data from the reference accession were aligned to the final assembly with an alignment rate of 99.7%. A KAT plot comparing the *k*-mer content of the assembly to that of short reads from the reference accession further suggests a high level of completeness (Supplementary Fig. 3). Heterozygosity can be observed from the first peak of the KAT plot as the proportion of *k*-mers absent from the assembly compared to the proportion of *k*-mers present, resulting from heterozygous loci being reduced into a single consensus assembly. Benchmarking Universal Single Copy Orthologs (BUSCO) (Manni et al. 2021) was used to assess the presence of conserved orthologs, and the results were indicative of a high-quality assembly with a score of 99.0% complete (C:99.0% [S:94.5%, D:4.5%], F:0.2%, M:0.8%, n:1614).

### Genome annotation

A custom repeat library was created for *S. microdontum* and used to mask the genome of repetitive regions. Repetitive sequences made up 65% of the genome, nearly half of which were identified as retroelements (32%) (Table 2, Supplementary Table 7). BRAKER2 and PASA2 were used to create the working model gene set, resulting in 69,964 working gene models; 63,256 were high-confidence gene models based on expression evidence, presence of a PFAM domain, not being a partial gene, and lack of internal stop codons. In total, 37,324 high-confidence genes were annotated (Table 2, Supplementary Table 8). Gene density tended to be highest toward the ends of the chromosomes except in the case of chromosome 2, which is acrocentric and contains the nucleolar organizing region (Fig. 1).

**Table 2.** Genome annotation metrics for the *S. microdontum* reference assembly.

Feature	Metric
Number of high-confidence genes	37,324
Number of high-confidence gene models	63,256
Mean gene length (bp)	1,549
Mean CDS length (bp)	1,186
Mean exon number	5
Number of repetitive elements	1,149,918
Length occupied by repetitive elements (bp)	504,536,055

Transposable elements were characterized using Extensive de-novo TE Annotator (EDTA) (Ou et al. 2019) (v2.2.2). A total of 744,506 transposable elements, making up 53.02% of the total genome sequence, were identified, comparable to the transposable element composition of other wild potato species (Aversano et al. 2015; Hosaka et al. 2022; Bozan et al. 2023; Supplementary Table 9). DNA transposons are less common than retrotransposons in the *S. microdontum* genome, comprising only 12.67% of the genome sequence compared to 39.99%. The majority of retrotransposons identified were classified as long terminal repeats; of these, unknown LTRs outnumbered Gypsy and Copia elements.

### Nucleotide-binding leucine-rich repeat genes

**Fig. 2.**
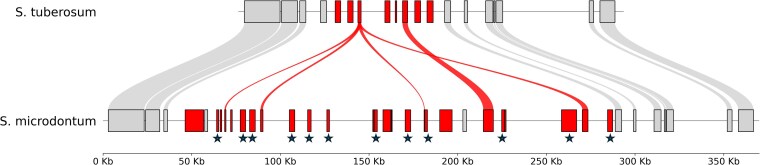
Local expansion of NLR genes in *S. microdontum*. Riparian plot showing syntenic region on chromosome 9 between *S. microdontum* and *S. tuberosum* reference genome DM v6.1. Each bar is a representative high-confidence gene model, and connecting ribbons represent synteny. NLR genes are colored red, and other genes are colored light gray. Stars represent NLR genes belonging to a single orthogroup unique to *S. microdontum* (Supplementary Table 12). Stars appearing slightly offset correspond to just a single gene (left or right, respectively) of a pair of small genes with little to no intervening sequence.

Nucleotide-binding leucine-rich repeat (NLR) genes are of particular interest to potato breeders as they are associated with disease resistance (Barragan and Weigel 2021). NLR-Annotator was used to predict NLR genes from the genome assembly of *S. microdontum* by searching for motifs associated with NLR genes. It should be noted that NLR-Annotator cannot differentiate between NLR genes that are tandemly duplicated with no intervening sequence. A total of 1,187 NLR genes were predicted, representing an enrichment compared to the 743 predicted from the *S. tuberosum* DM v6.1 reference genome and the 741 predicted on average per haplotype from *S. tuberosum* var. Atlantic (2,963 total across 4 haplotypes) (Supplementary Tables 10 and 11). Results were assessed for overlaps with the *S. microdontum* genome annotation, with a total of 1,143 predicted NLR genes (96.3%) intersecting a representative working gene model and 1,105 predicted NLR genes (93.1%) intersecting a representative high confidence gene model. Like overall gene density, the density of predicted NLR genes increases toward the ends of chromosomes; however, the total number of NLR genes per chromosome varied widely (Fig. 1). Chromosome 3 was predicted to have the fewest NLR genes (11), while chromosome 11 had the most (281). There are several regions of particularly high NLR gene density, such as one locus on chromosome 9 with 25 NLR genes in a span of ca. 250 kb (Fig. 2) (Supplementary Table 12). Twelve of these belong to a single orthogroup not represented in the *S. tuberosum* reference assembly DM v6.1, and 4 others are duplications of an NLR gene found in a single copy in DM. Heterogeneous NLR clusters such as this have been known to arise through a combination of tandem duplications, ectopic duplications, and unequal recombination events that result in a complex mixture of genes from different lineages (Leister 2004). This NLR cluster and others like it in the *S. microdontum* genome could be attractive breeding targets due to the abundance of NLR genes and relative lack of other genes that could be associated with undesirable traits.

### Comparative genomics among wild potato species

**Fig. 3.**
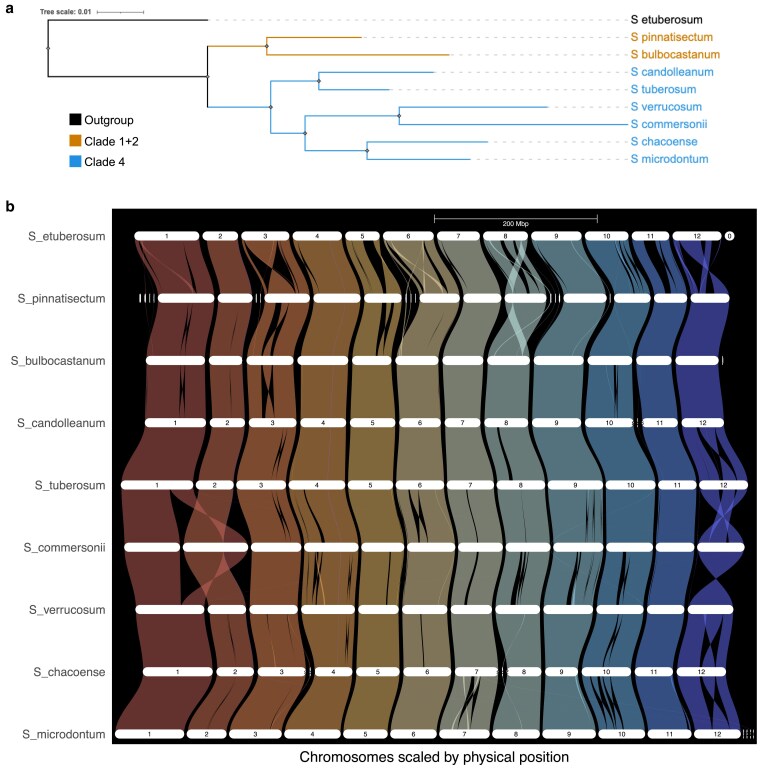
Phylogenetic relationships and genome comparison of *S. microdontum* with other wild potato species. a) Species tree of *S. microdontum*, wild potato relatives, and the *S. tuberosum* reference genome DM v6.1, colored by clade membership according to taxonomic groupings in the literature (Spooner and Castillo 1997; Hardigan et al. 2015; Spooner et al. 2018; Huang et al. 2019). b) Riparian plot displaying syntenic relationships between *S. microdontum*, 7 wild potato species, and *S. tuberosum*.

GENESPACE was used to compare the genome of *S. microdontum* with the chromosome-scale genome assemblies of 6 other wild potato species as well as *S. tuberosum* and a more distantly related nontuber-bearing wild potato species *Solanum etuberosum* (Supplementary Table 4). Species were chosen for synteny analysis based on the presence of a chromosome-scale publicly available genome assembly. These species are diverse in their origins, spanning from North-Western Mexico to Central Chile and possess a range of resistances to major potato diseases such as late blight, potato leaf roll virus, and bacterial wilt (*Ralstonia solanacearum* Smith) (Ruiz de Galarreta et al. 1998; Micheletto et al. 2000; Carputo et al. 2009). GENESPACE utilizes the output of OrthoFinder, which detects orthologous genes across species. OrthoFinder identified 14,843 orthogroups that included all species, and the resulting orthologous gene trees were used to create a species tree (Fig. 3a). The tree was colored according to taxonomic classifications from the literature that generally group wild potato species into 4 distinct clades, with clade 4 being the largest and inclusive of cultivated potato species (Spooner and Castillo 1997; Hardigan et al. 2015; Spooner et al. 2018; Huang et al. 2019). The species tree is in agreement with previous taxonomic classifications, placing *S. microdontum* in a clade with *S. chacoense, S. commersonii,* and *S. verrucosum*.

A riparian plot visualizing genome synteny and structural variation across the 9 species was also generated via GENESPACE (Fig. 3b). Overall, genomic structure is very well conserved among the 9 species with a few exceptions. *S. etuberosum* displayed the highest level of structural rearrangements owing to its more distant evolutionary relationship. Across the rest of the species, a series of inversions can be observed on chromosome 12 that could potentially indicate a conserved rearrangement pattern among several lineages. The presence of a large rearrangement overlapping the centromere has been previously described between *S. verrucosum* and *S. tuberosum* on chromosome 12, and segregation distortion has been known to occur as a result (Hosaka et al. 2022). This aligns with recent *Solanum* pan-genome studies showing that while a high level of macrosynteny is maintained across *Solanum* and especially within section *Petota*, lineage-specific structural variants, gene duplications, and paralogue diversification contribute to diversity important for adaptation and breeding (Wu et al. 2023; Benoit et al. 2025). These results serve to contextualize the *S. microdontum* reference genome within a clade of closely related wild potato species and provide a point of reference for further studies of *Solanum* species evolution involving *S. microdontum*.

### Diversity panel

The *S. microdontum* diversity panel contains 107 accessions of *S. microdontum* obtained from USDA-GRIN, originating from Bolivia and Argentina and spanning much of the native range of the species (Correll 1962) (Supplementary Table 1). Publicly available Illumina short reads from the *S. tuberosum* reference genotype, DM, were included to allow for comparison between *S. microdontum* accessions and cultivated potato. Following read cleaning, an average of 15× sequence coverage per *S. microdontum* accession was obtained with the exception of the reference accession PI 595506, which was sequenced to a coverage of 70× (Supplementary Table 2).

**Fig. 4.**
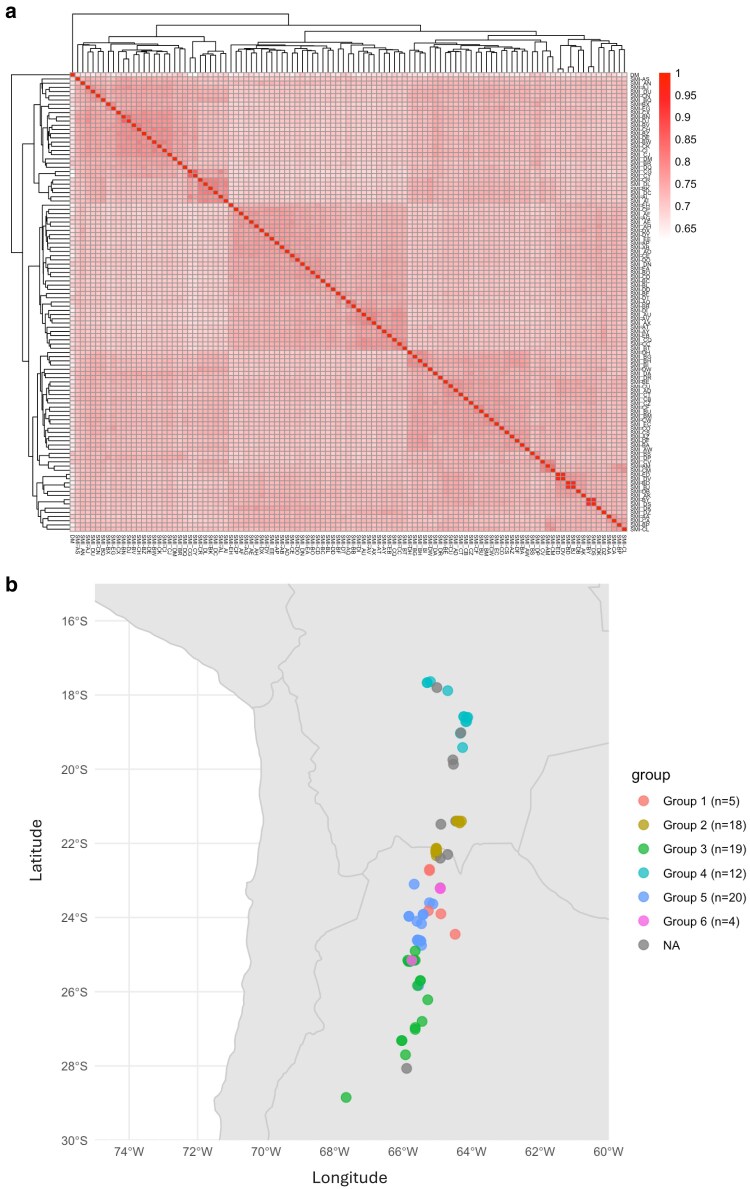
Kinship among *S. microdontum* diversity panel members. a) Kinship heatmap. Each PI is represented by one row and one column. Higher kinship values correspond to increased relatedness as suggested by a *k*-mer analysis. b) Collection coordinates colored by population group. *N* refers to the number of individuals visualized per group, which may be less than the number of individuals placed in that group based on their kinship values, due to 19 accessions lacking collection coordinates.

A *k*-mer-based kinship matrix describing relatedness between members of the diversity panel was generated from the reads using the kmerGWAS pipeline (Voichek and Weigel 2020) and visualized in the form of a principal component analysis, heatmap, and a dendrogram (Fig. 4a, Supplementary Figs 4 and 5). Underlying population structure can be observed from the heatmap, where the darker portions of the map indicate higher levels of relatedness (Fig. 4a). Inter-relatedness was queried systematically by setting a threshold kinship value and finding groups of individuals whose kinship exceeded the threshold. This can be visualized graphically, in which each individual is represented by a node and each kinship value exceeding the threshold is an edge connecting 2 nodes (Supplementary Fig. 6). Within a group, each node is accessible from every other node, either directly or indirectly. This yielded a total of 16 groups, 6 of which contained 5 or more members. Each accession with available coordinate data was plotted on a map according to where that PI was collected and colored according to their membership in the 6 largest groups. This revealed a clear correlation between geographic origin and population structure, where individuals from similar latitudes tend to be more closely related (Fig. 4b). These results suggest that *S. microdontum* accessions from similar latitudes may also be more similar in traits such as disease resistance, agronomic performance, and tuber morphology, all of which are key factors that influence breeders’ decisions when selecting accessions for introgression or trait screening.

## Conclusions

Here, we present a chromosome-scale reference genome for the wild potato species *S. microdontum*. It is highly contiguous and complete, demonstrated by a variety of scoring methods such as BUSCO and LAI, *k*-mer-based visualization, and comparison to other wild and cultivated potato genomes. A total of 37,324 high-confidence genes were identified, and 65% of the genome was found to be composed of repetitive sequence content, of which 53% is transposable elements. The reference accession contains over 1,000 NLR genes, which are of interest to potato breeders looking to incorporate disease resistance into their programs. Kinship among a diversity panel of *S. microdontum* accessions was correlated with geographic latitude, which may also be of interest to potato breeders choosing an accession for introgression. The genetic resources presented here will facilitate the identification and study of adaptive traits in *S. microdontum* and the ease with which they can be introgressed into cultivated potato breeding programs.

## Supplementary Material

jkaf253_Supplementary_Data

## Data Availability

All raw sequencing data have been deposited into the National Center for Biotechnology Information Sequence Read Archive under BioProject Number PRJNA1290315. This Whole Genome Shotgun project has been deposited at DDBJ/ENA/GenBank under the accession JBPVVW000000000. The version described in this paper is version JBPVVW010000000. Large datasets (assembly and annotation) are also available via figshare at https://doi.org/10.6084/m9.figshare.29546450. Supplemental material available at G3 online.
