# Supplementary material for: Building genomic resources to facilitate the study and use of Solanum microdontum, a wild relative of cultivated potato
Source: G3 (Bethesda). 2025 Oct 23;16(1):jkaf253. doi: 10.1093/g3journal/jkaf253 (PMC12774605; doi:10.1093/g3journal/jkaf253)
Supplement: jkaf253_Supplementary_Data [file jkaf253_supplementary_data.zip › Supplemental_Figure_and_Table_Legends_G3-2025-406174.docx]

**Supplemental Figure S1: Estimation of heterozygosity in the reference accession (PI 595506) using GenomeScope.** Abbreviations are len: inferred total genome length; uniq: percent of the genome that is unique (not repetitive); het: overall rate of heterozygosity; kcov: mean kmer coverage for heterozygous bases; err: error rate of the reads; dup: average rate of read duplications.

**Supplemental Figure S2: Hi-C contact map.** Blue boxes indicate pseudomolecules, green boxes indicate individual contigs. Red color intensity indicates density of contacts identified.

**Supplemental Figure S3: Genome completeness and heterozygosity as estimated by KAT.** Color legend refers to number of times a *k*-mer appears in the final consensus assembly.

**Supplemental Figure S4: Dendrogram of diversity panel members.**

**Supplemental Figure S5: Principal component analysis plot of diversity panel members**

**Supplemental Figure S6: Diversity panel group membership graph visualization**
