# Supplementary figures and images for: Building genomic resources to facilitate the study and use of Solanum microdontum, a wild relative of cultivated potato
Source: G3 (Bethesda). 2025 Oct 23;16(1):jkaf253. doi: 10.1093/g3journal/jkaf253 (PMC12774605; doi:10.1093/g3journal/jkaf253)

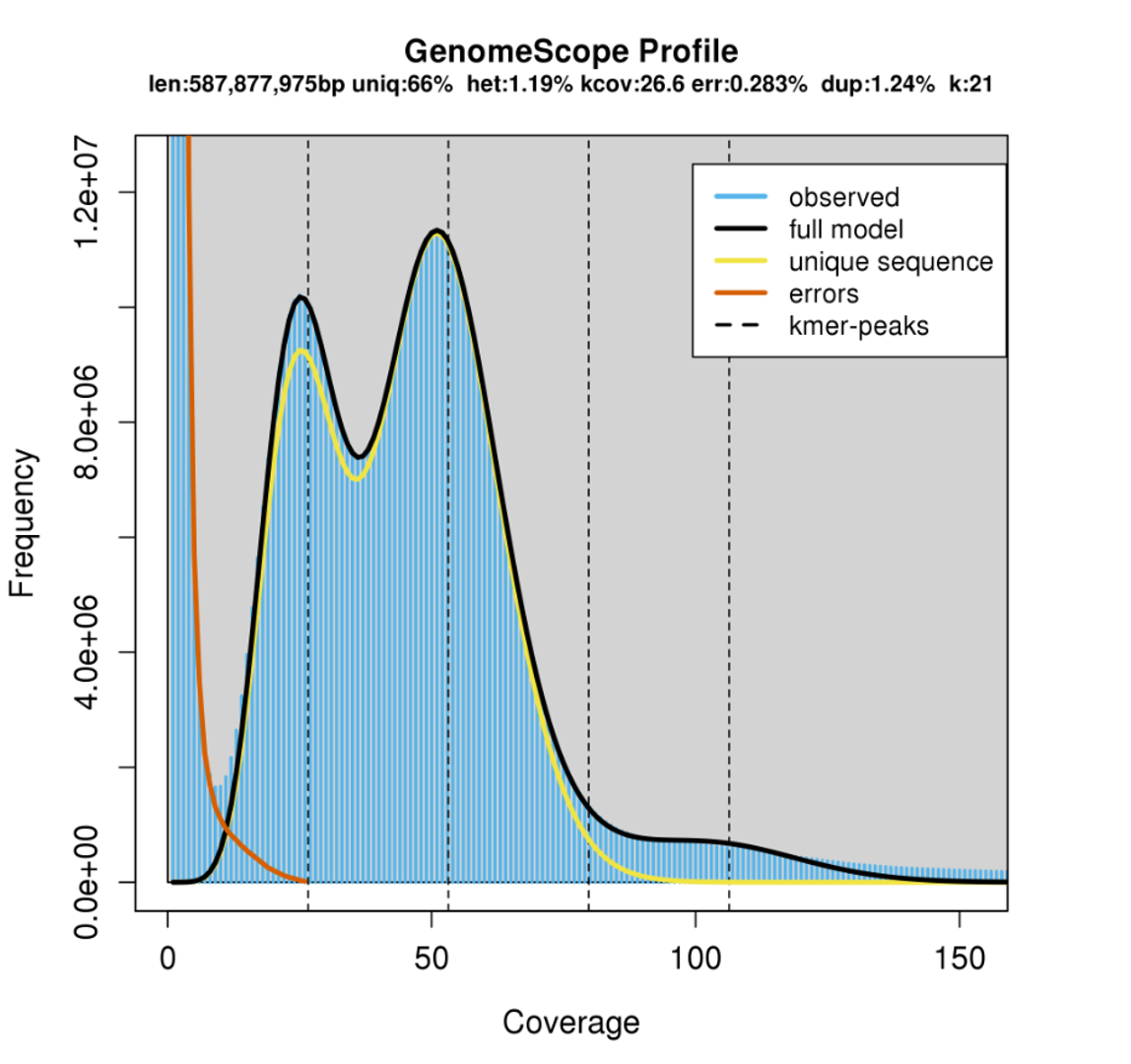

Supplement: jkaf253_Supplementary_Data [file jkaf253_supplementary_data.zip › Supplemental_Figure_1_G3-2025-406174.png]

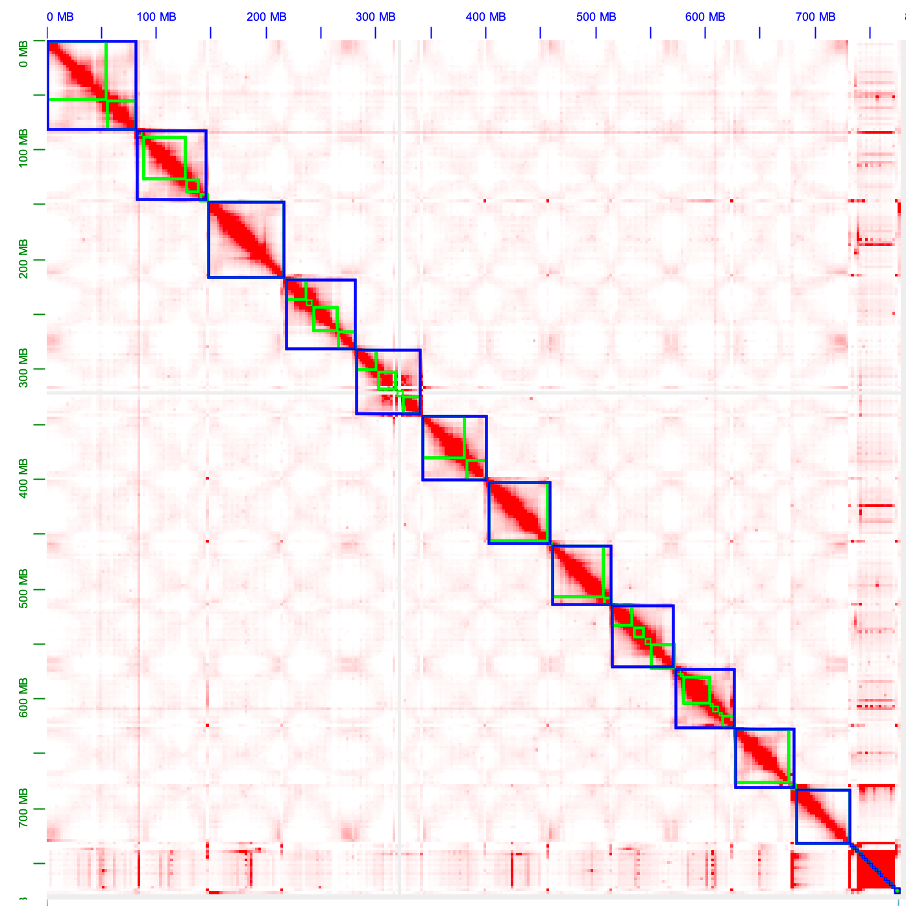

Supplement: jkaf253_Supplementary_Data [file jkaf253_supplementary_data.zip › Supplemental_Figure_2_G3-2025-406174.png]

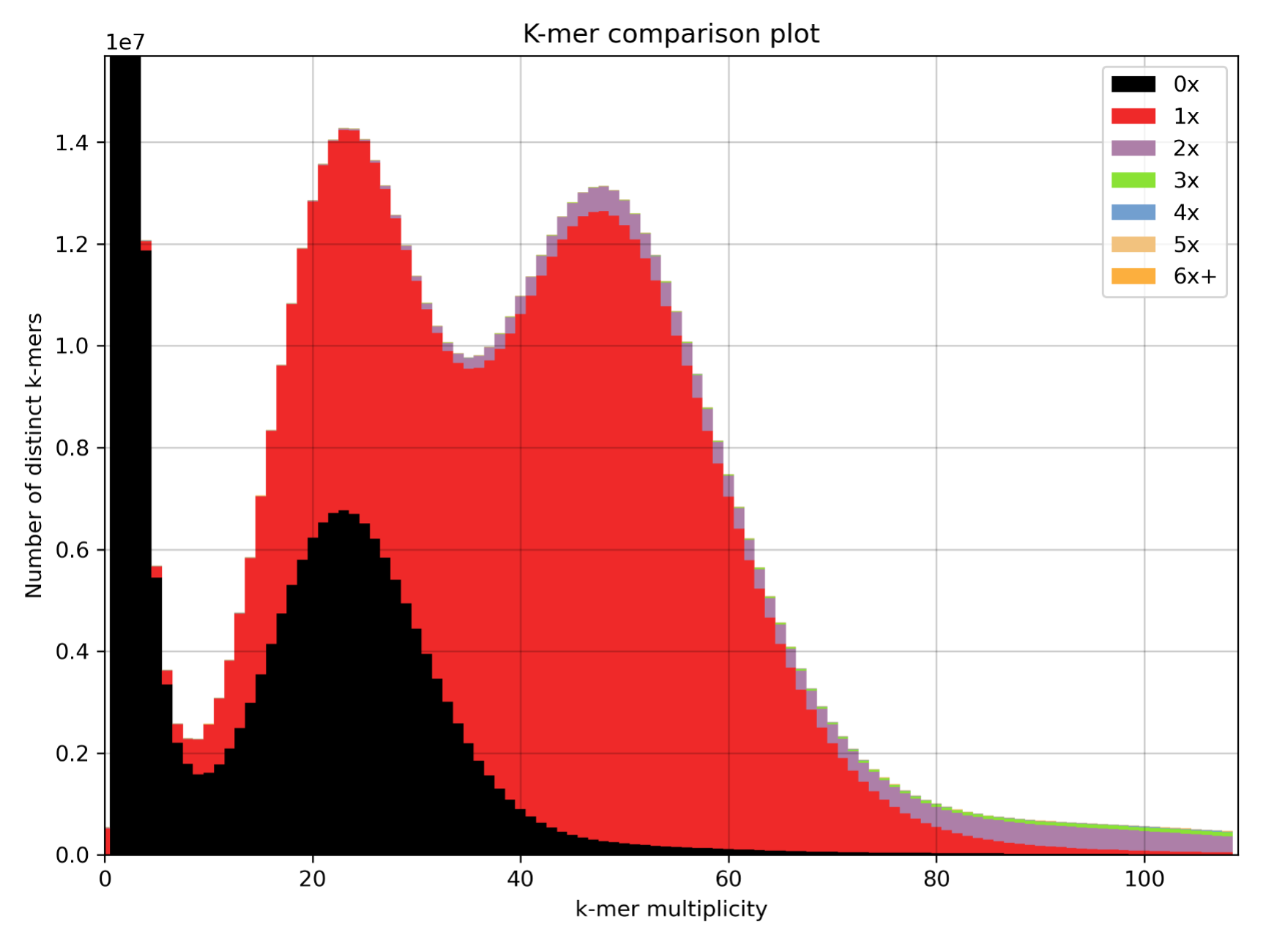

Supplement: jkaf253_Supplementary_Data [file jkaf253_supplementary_data.zip › Supplemental_Figure_3_G3-2025-406174.png]

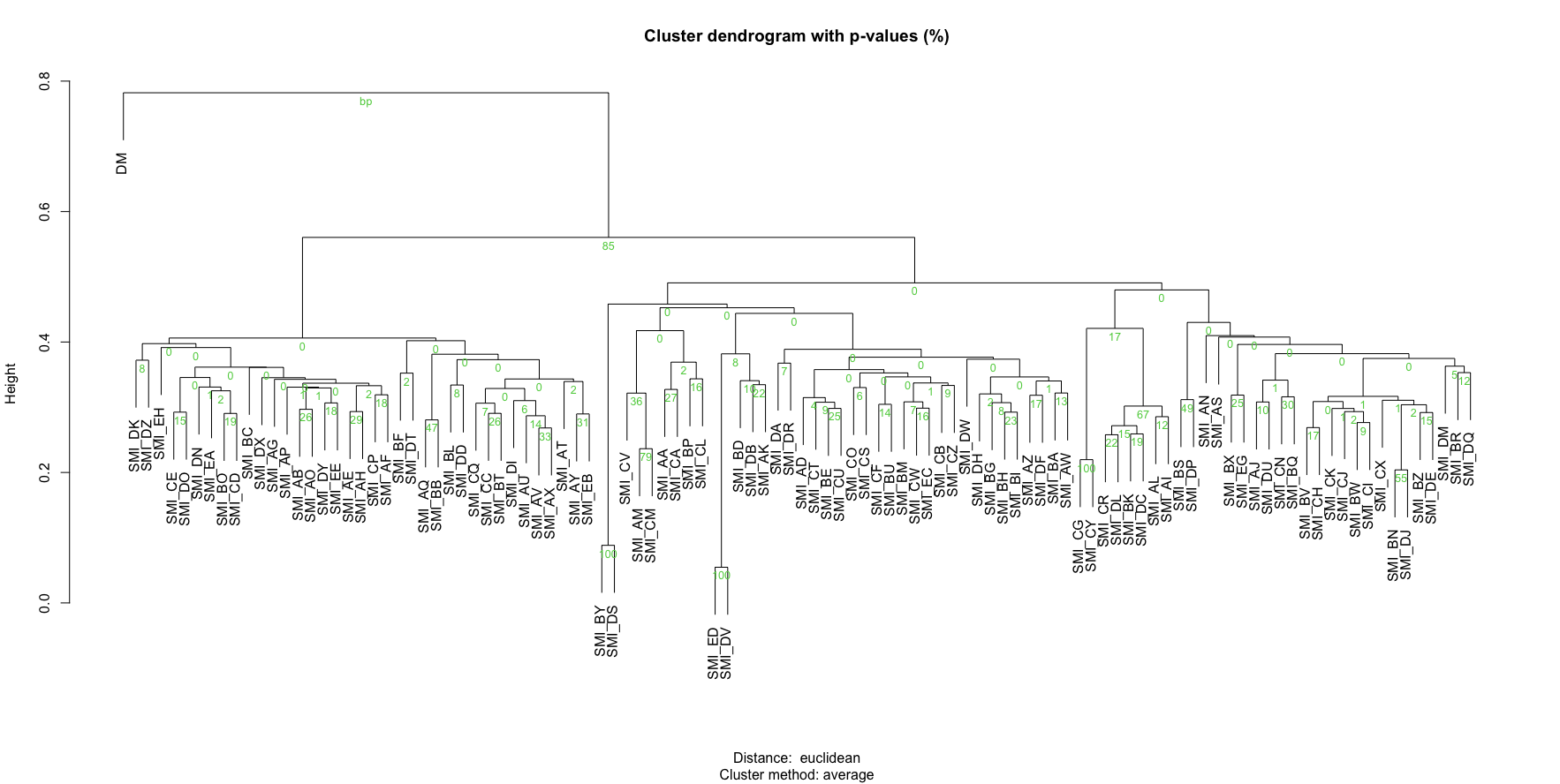

Supplement: jkaf253_Supplementary_Data [file jkaf253_supplementary_data.zip › Supplemental_Figure_4_G3-2025-406174.png]

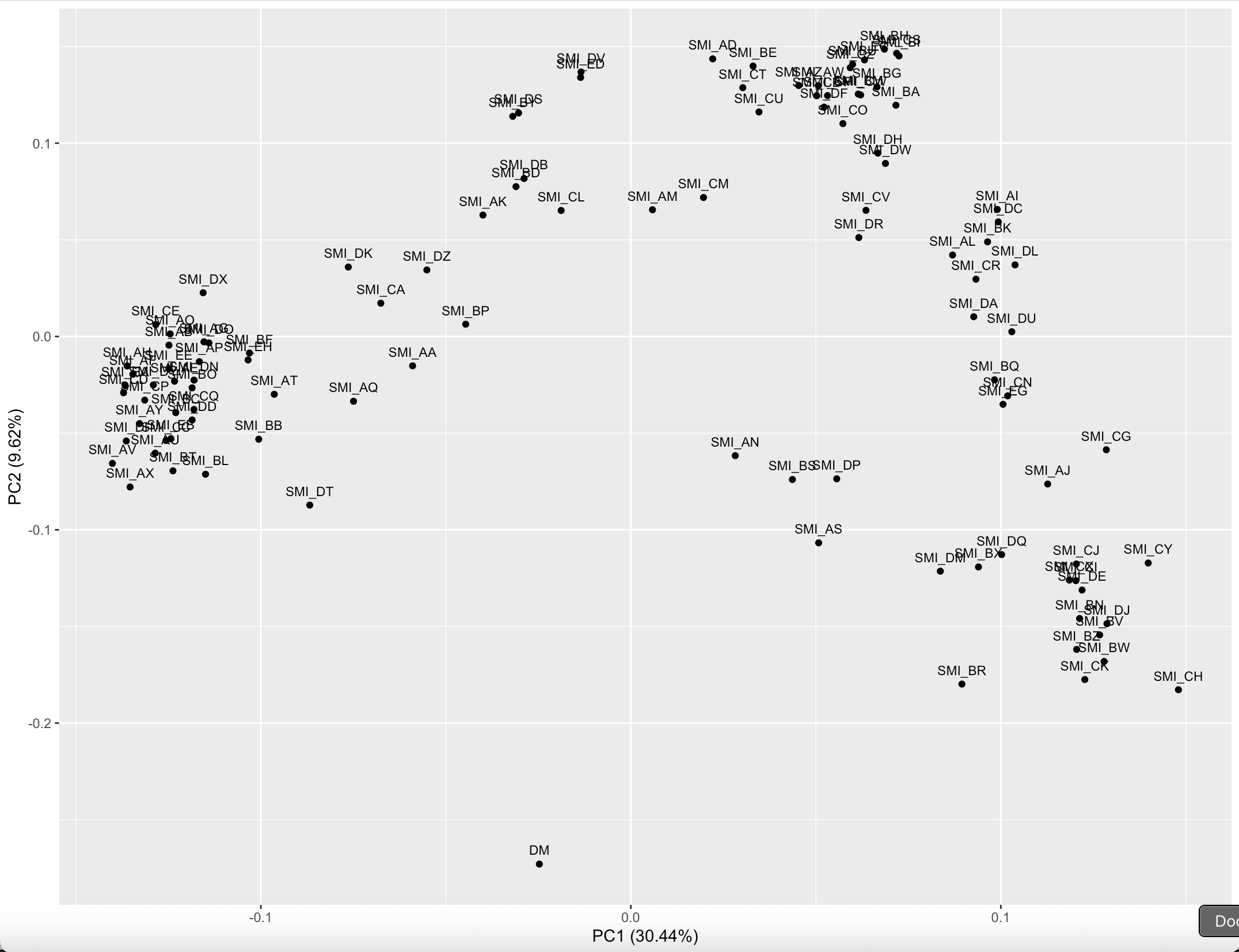

Supplement: jkaf253_Supplementary_Data [file jkaf253_supplementary_data.zip › Supplemental_Figure_5_G3-2025-406174.png]

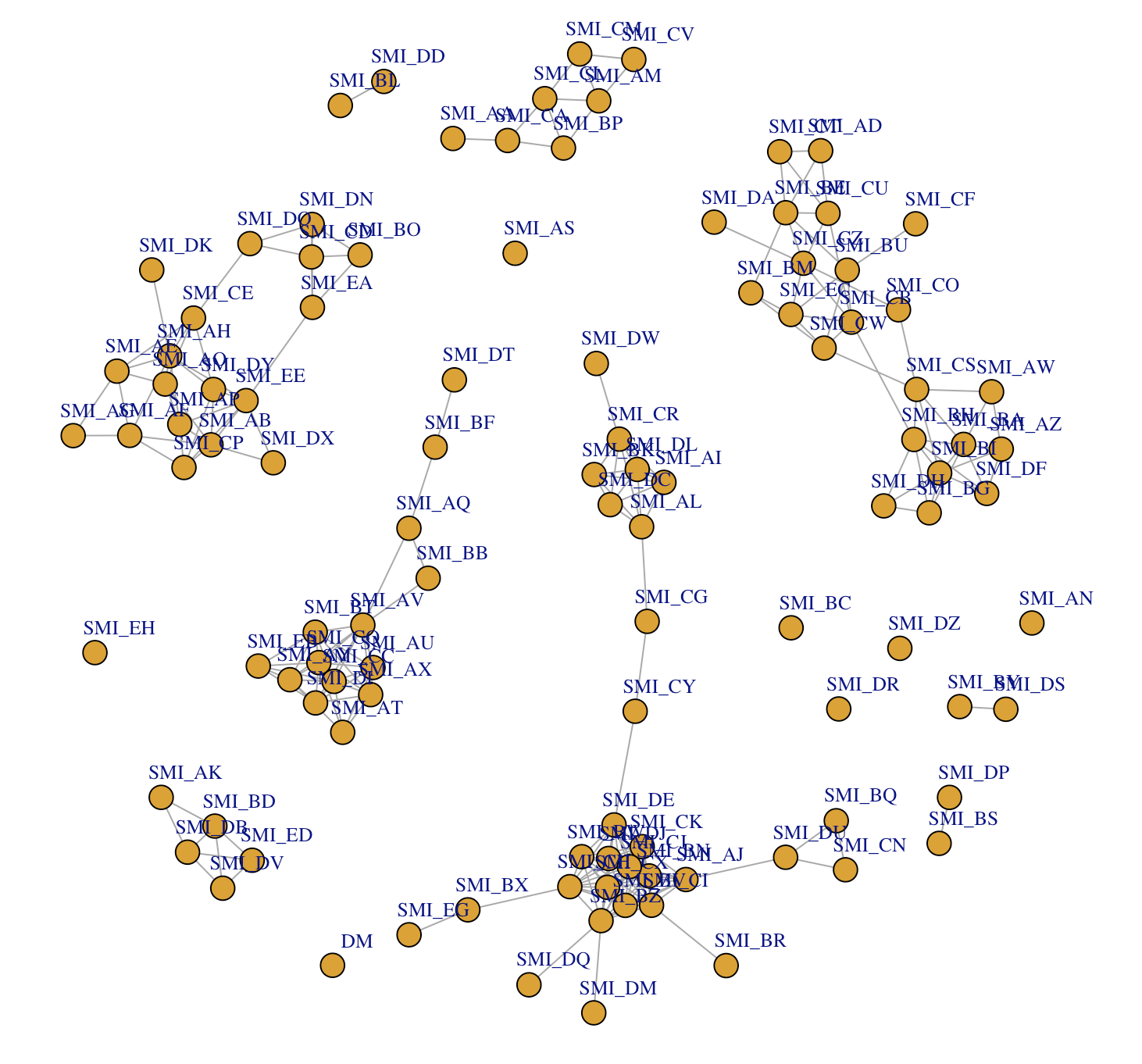

Supplement: jkaf253_Supplementary_Data [file jkaf253_supplementary_data.zip › Supplemental_Figure_6_G3-2025-406174.png]
